# Presence of entry receptors and viral markers suggest a low level of placental replication of hepatitis B virus in a proportion of pregnant women infected with chronic hepatitis B

**DOI:** 10.1038/s41598-022-22699-8

**Published:** 2022-10-22

**Authors:** Garima Garg, M. N. Meenu, Kajal Patel, Ravinder Singh, Priyal Gupta, Shashank Purwar, Sramana Mukhopadhyay, Nitu Mishra, Sudheer Gupta, Sumit Kumar Rawat, Harsh Goel, Rahul Kumar, Pranay Tanwar, Jitendra Singh, Shashwati Nema, Debasis Biswas, Nirupma Trehanpati, Anirudh K. Singh, Ashish Kumar Vyas

**Affiliations:** 1 Department of Microbiology, All India Institute of Medical Sciences, Bhopal, India; 2 Department of Molecular and Cellular Medicine, Institute of Liver and Biliary Sciences, New Delhi, India; 3 Department of Pathology, All India Institute of Medical Sciences, Bhopal, India; 4 Department of Gynecology and Obstetrics, Gandhi Medical College, Bhopal, India; 5 Department of Microbiology, Bundelkhand Medical College, Sagar, India; 6 Laboratory Oncology Unit, Dr. B.R.A. Institute Rotary Cancer Hospital (Cancer Centre), All India Institute of Medical Sciences, New Delhi, India; 7 Department of Translational Medicine Centre, All India Institute of Medical Sciences, Bhopal, India

**Keywords:** Diseases, Gastroenterology, Medical research

## Abstract

The transplacental route of vertical transmission of Hepatitis B Virus (HBV) has been known for over a decade. Here we present evidence which suggest HBV can replicate in placenta. Forty-one HBsAg positive and 10 control pregnant women were enrolled in the study after obtaining informed consent. HBV positives were further divided in the High Viral Load (HVL) Group and Low Viral Load (LVL) Group according to INASL guidelines 2018. The Presence of the HBV DNA and expression of NTCP in the placenta was analyzed by qPCR/RT-qPCR and/or immunohistochemistry (IHC). The presence of cccDNA was assessed using Digital Droplet PCR while the presence of pre-genomic (pg) RNA was assessed through qRT-PCR and sequencing. The presence of HBeAg and HBcAg in the placenta was assessed by IHC. Immunostaining of NTCP, HBeAg and HBcAg on trophoblasts along with the presence of total HBV DNA, cccDNA and pgRNA indicated, that these cells are not only susceptible to HBV infection but may also support viral replication. This is further supported by the finding that trophoblasts of the several HBeAg seronegative samples harbored the HBeAg. Although, we did not find any correlation in NTCP expression and viral markers with viral load indicates placental replication may not aping hepatocytes. The presence of the HBV receptor, NTCP along with the presence of cccDNA, pgRNA, and HBeAg in placenta of HBV infected females without circulating HBeAg suggest that placenta act as a replication host.

## Introduction

Chronic Hepatitis B (CHB) infection is a major global health problem with 290 million infected people worldwide resulting in 887,000 deaths annually^1^. Vertical transmission of HBV is a leading cause of chronicity^2^. About 90–95% children acquiring infection from mother develop chronicity compared to immunocompetent adults where this risk is just 5–10%. The vertical transmission is the main route of infection in developing countries like India where 1–4% of population is carrier of HBV. Patients with CHB have high risk of developing end-stage cirrhosis and hepatocellular carcinoma^3^.

Despite being the key driver of HBV chronicity, mechanism of vertical transmission of HBV remains elusive. Three modes of vertical transmission; intrauterine or prenatal, natal and postnatal have been proposed^4^ with none of them providing conclusive evidence to support a specific route.

Other viruses such as Human Immunodeficiency Virus-1 (HIV-1) and Cytomegalovirus (CMV) have been shown to be transmitted to the newborns through placenta^5^. However, placenta mediated transmission mechanism(s) for HBV are understudied. Findings from our previous studies as well as from others indicated, HBV may replicate in placenta^6,7^. In a study examining the association of maternal immunity and viral load with vertical transmission of HBV, we observed the expression of HBV co-receptor, Asialoglycoprotein (ASGPR) on placental cells^6^. Furthermore, Bhat et al. showed the replication of HBV in immortalized trophoblasts^7^. While these observations hint at the possibility of placenta being an additional organ supporting the HBV replication, evidences for in vivo replication in human placenta are not yet produced^7,8^.

In this study, using blood and placenta samples from HBV infected and healthy controls, we for the first time, provide experimental evidence suggesting that HBV can enter placenta and replicate there, which may facilitate viral transmission.

## Results

### Virological and serological characteristics

The demographic and serological profile of subjects enrolled in the study is shown in Table 1. We did not find difference in base line clinical parameter between the HVL and LVL groups. Peripheral HBeAg analysis showed 26.66% and none were positive in HVL and LVL groups, respectively.

**Table 1:** Demographic and serological characteristics of study participants:

Parameter	Control (n = 10)	LVL (n = 19)	HVL (n = 22)
Age (years)	23.9 ± 3.755	22.47 ± 2.54	24.51 ± 3.17
HBV DNA (IU/ml)	–	39.2 (3.58–1790)	3.0 × 10^6^ (2.2 × 12^3^–4.78 × 10^8^)
AST (IU)	19.9 ± 15.75	99.81 ± 67.57	105.6 ± 95.85
ALT (IU)	29.51 ± 12.24	78.38 ± 37.48	56.71 ± 34.28
HBeAg	–	NIL	26.66%

Age, mean ± SD; HBV DNA, median ± range; Aspartate Aminotransferase (AST), mean ± SD; Alanine Aminotransferase (ALT), mean ± SD.

### Placenta of HBV positive patients contains viral components

**Figure 1:**
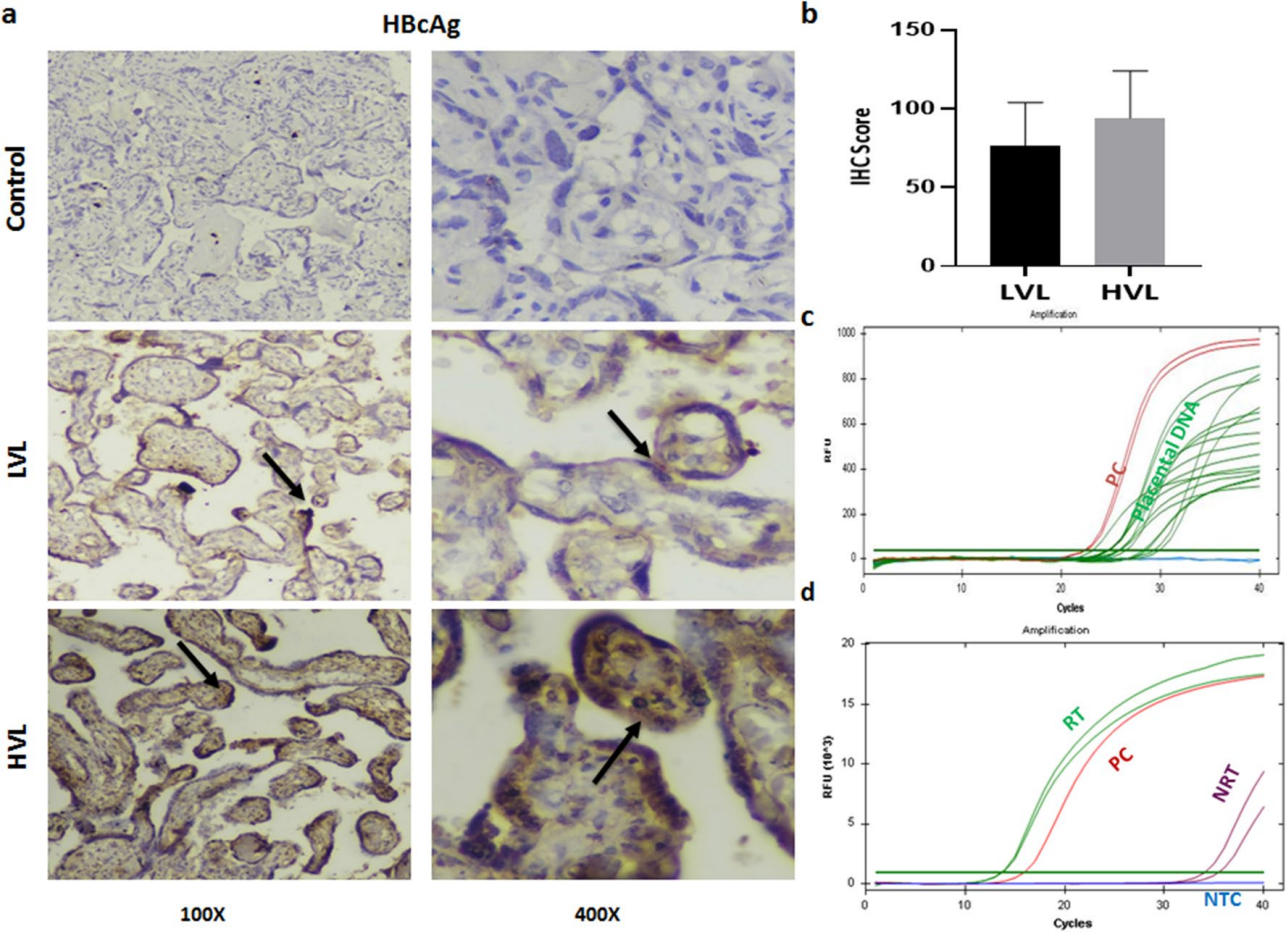
Presence of viral markers in placenta. (**a**) Representative figure showing the presence of HBcAg in placenta detected through Immunohistochemistry (IHC), cytoplasmic staining in LVL and HVL groups was observed, and no staining in control. (**b**) IHC Scores of HVL and LVL groups showed no significant difference (HVL 94.62 ± 9.46 vs. LVL 76.5 ± 8.68 p = 0.9253), p-value was calculated for a risk threshold α = 0.05, p* < 0.05. (**c**) Detection of total HBV DNA in placenta through qPCR amplification and (**d**) detection of pre-genomic RNA in placenta through qRT-PCR amplification. *HVL* High Viral Load Group, *LVL* Low Viral Load Group, *PC* Positive Control, *NTC* No Template Control, *RT* cDNA prepared with Reverse Transcriptase, *NRT* cDNA prepared without Reverse Transcriptase.

Earlier studies have demonstrated the presence of HBsAg and HBcAg in placenta^9,10^. The status of HBcAg in placental cells was determined using IHC and it was found in 70% (7/10) samples of HVL group and 80% (8/10) samples of LVL group (Fig. 1a). We did not find any significant difference in the IHC score of HBcAg in HVL and LVL groups (HVL 94.62 ± 9.46 vs. LVL 76.5 ± 8.68, p = 0.9253) (Fig. 1b). No staining in the control placenta ruled out non-specific staining of HBcAg in HBV positive placenta. Presence of HBV in placental cells was further assessed by detection of viral DNA using qPCR. Conserved regions of X and Core ORF were targeted using specific primers and were detected in 90% (9/10) of HVL and 71.12% (5/7) of LVL samples (Fig. 1c, Supplementary Fig. 1). The high percentage of HBV DNA and/or HBcAg positivity in both groups indicated placental infection in most of the samples. We next investigated if placenta can act as reservoir for HBV replication.

### HBV replicates in placental cells

Presence of viral components in the placenta could be due to; (1) translocation of viral components from peripheral blood, or (2) the virus replicates in the placenta.

#### Nucleic acid markers for HBV replication

**Figure 2:**
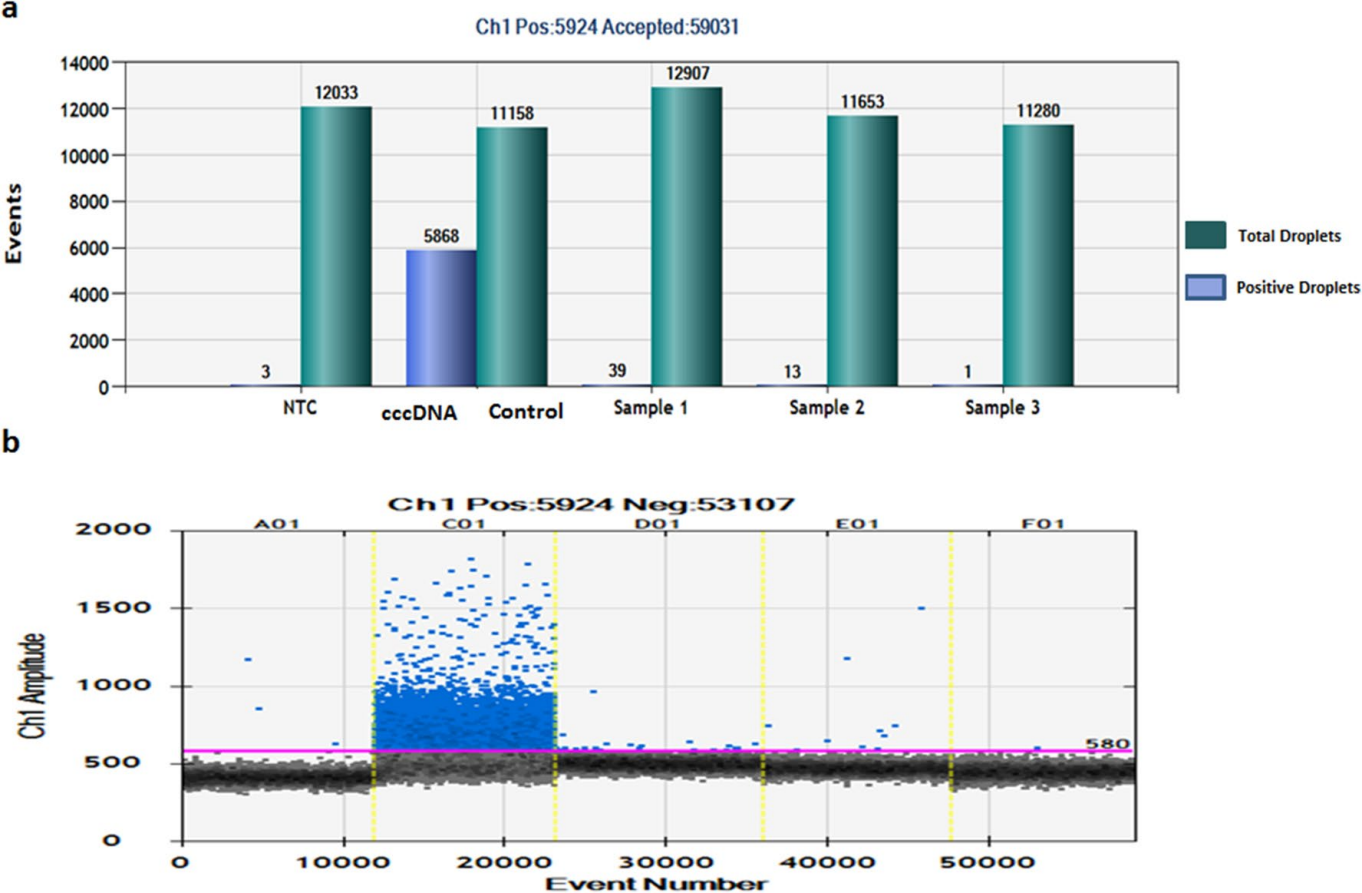
Presence of cccDNA in placenta. (**a**) cccDNA was detected through fluorescence amplitudes of HBV in the infected samples using Digital Droplet PCR (ddPCR), Absolute quantification of cccDNA showed more than 11,000 events generated in which, the highest positive droplets were found in positive control isolated from HepAD38 cells^11^, while in Sample 1 (S1), Sample 2 (S2), Sample 3 (S3) and NTC; 39, 13, 1 and 3 positive droplets were observed, respectively. We consider sample positive for cccDNA if more than 3 droplets are detected. (**b**) Plot showing threshold fluorescence amplitude and droplet count.

Pregenomic RNA (pgRNA) serves as a template for HBV replication and its presence in cells is used as a marker for viral replication. We assessed 3 high viral load samples for the presence of pgRNA using specific primers and observed positive amplification in two samples which was further confirmed by Sanger sequencing. (Fig. 1d, Supplementary Fig. 2). We further assessed the presence of cccDNA, another critical marker for viral replication and transcription using Digital Droplet PCR (ddPCR). Exonuclease treated placental DNA samples were amplified using specific primers and probe. A sample giving more than three positive droplets is considered positive for the template in ddPCR. While no template control (NTC) gave three positive droplets, two out of the three placental samples gave 39 and 13 positive droplets confirming the presence of cccDNA in placenta (Fig. 2).

#### Protein marker for HBV replication

**Figure 3:**
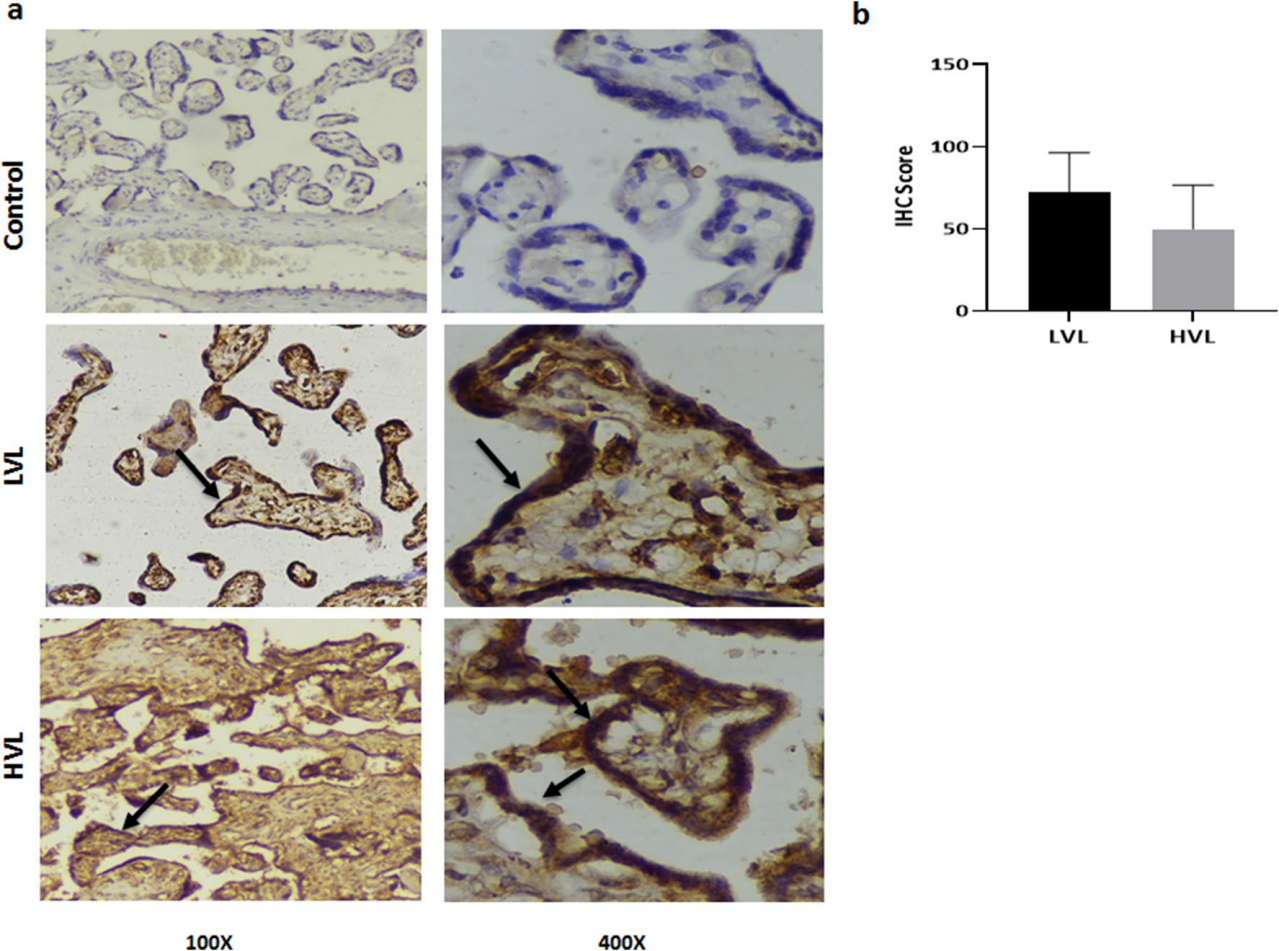
Presence of HBeAg in placenta. (**a**) Representative image showing the presence of HBeAg in placenta by Immunohistochemistry, no staining in control and strong cytoplasmic staining in LVL and HVL groups was observed. (**b**) IHC Scores of HVL and LVL groups showed no significant difference (HVL 50 ± 8.43 vs. LVL 72.5 ± 7.51 p = 0.2204; * < 0.05). Two-tailed p-value was calculated for a risk threshold α = 0.05. p* < 0.05. *HVL* High Viral Load Group, *LVL* Low Viral Load Group.

HBeAg is a protein signature of HBV replication. HBeAg was detected in peripheral blood and placental tissue of the enrolled subjects using ELISA and IHC, respectively. We found only 7.14% (1/14) were positive for both while 14.28% (2/14) were positive for HBeAg in circulation but not in placenta and 28.57% (4/14) did not show any detectable levels of HBeAg in the circulation as well as in placenta. Surprisingly, HBeAg was detected in 50% (7/14) of the placental samples where cognate plasma samples tested negative for the antigen, showing not all the placental positivity for HBeAg could be attributed to the peripheral levels and is most likely a result of viral replication in these cells. While we observed cytoplasmic staining in the trophoblasts of 40% (4/10) in HVL group and 80% (8/10) in LVL group (Fig. 3a), no statistically significant difference was observed is IHC scores of HVL and LVL groups (HVL 50 ± 8.43 vs. LVL 72.5 ± 7.51, p = 0.2204; * < 0.05) (Fig. 3b). To eliminate any false positivity, we did not consider weak intensity staining as positive. There was no staining in the control group placentas showing specific immunostaining.

This observation along with the presence of cccDNA and Pre-genomic RNA in the placenta strongly suggests, this organ acts as a reservoir.

### Placental cell express NTCP, the entry receptor of HBV

**Figure 4:**
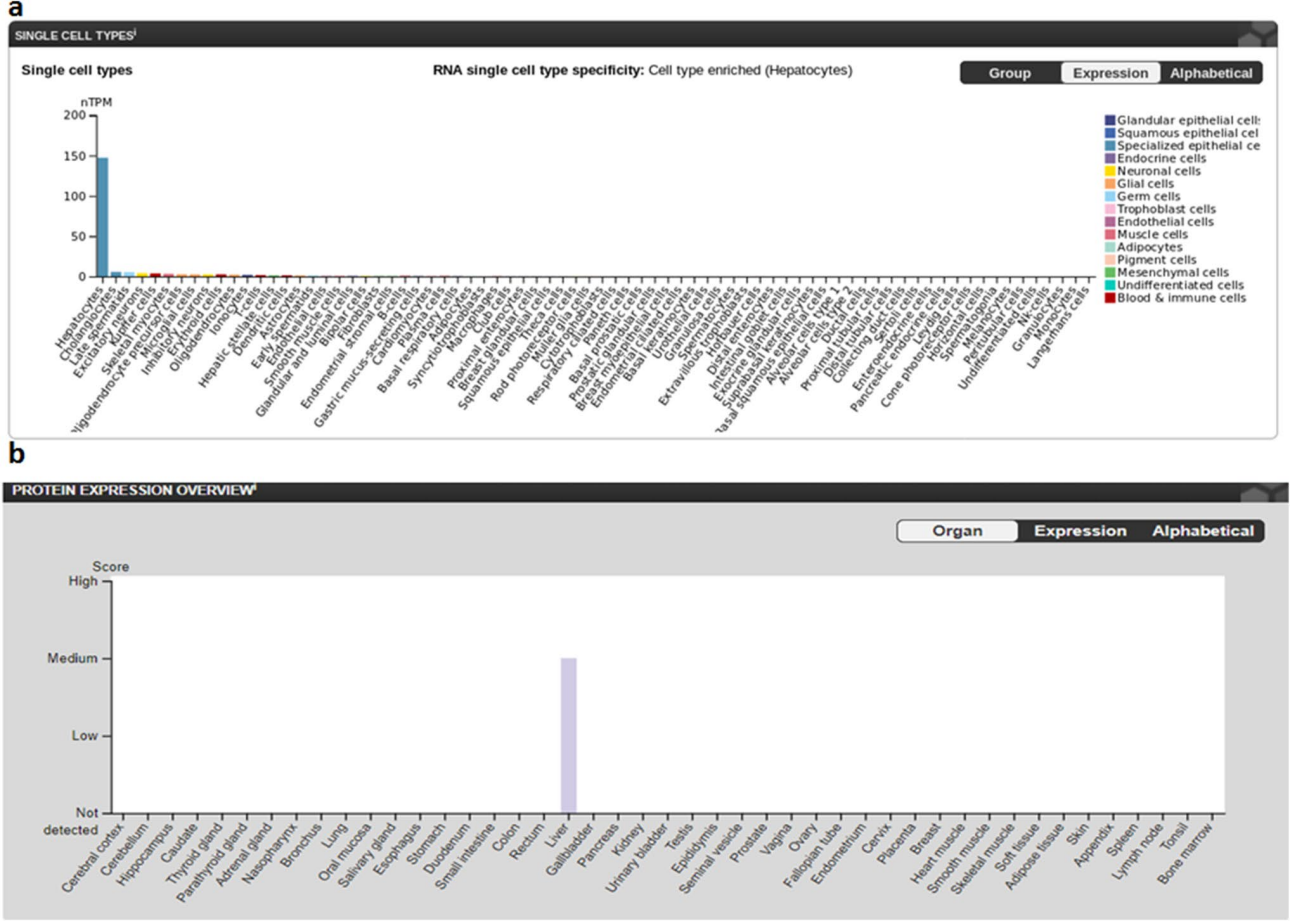
Expression of NTCP indifferent tissues/cells. (**a**) Consensus normalized RNA expression and protein expression overview in different cell types. (**b**) Color-coding in the plot, is based on cell type groups, each consisting of cell types with functional features in common.

We postulated that placenta may act as a reservoir and facilitate viral replication. For this to happen, placenta must express entry receptor(s) for HBV. NTCP is the primary receptor for virus on hepatocytes and is thought to be exclusive to these cells^12^. We looked for the expression data of NTCP in different tissues/cells available in "The Human Protein Atlas" (http://www.proteinatlas.org). Expression of NTCP in healthy human cell types is reported mainly in Hepatocytes and to a lower level in Cholangiocytes, Late spermatids, Excitatory neurons, Kupffer cells, Skeletal myocytes, Oligodendrocyte precursor cells, Microglial cells, Erythroid cells, Inhibitory neurons, Oligodendrocytes, Ionocytes, T-cells, Dendritic cells, Hepatic stellate cells, Astrocytes, Early spermatids, Endothelial cells, Smooth muscle cells and B-cells. However, cells specific to placenta were not reported to express NTCP. The consensus normalized expression of NTCP for single cell RNA (nTPM) and protein expression overview from all cell types are shown in Fig. 4a,b, respectively.

**Figure 5:**
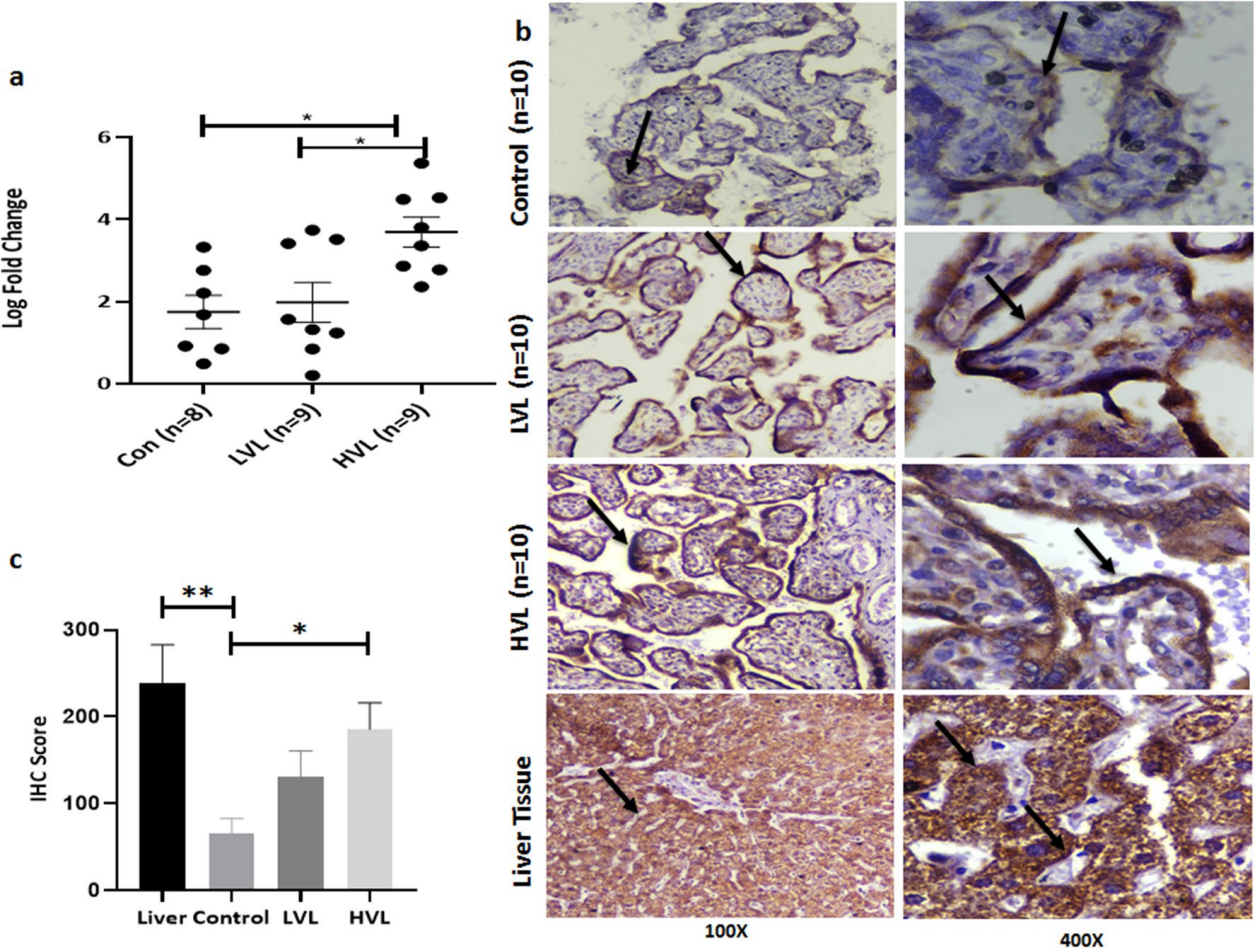
Expression of NTCP in placenta. (**a**) NTCP expression in placenta samples from various groups was measured using qRT-PCR. It was significantly up regulated in HVL group when compared with control group (HVL 3.69 ± 0.36 vs. Control 1.74 ± 0.40, p = 0.0117; * < 0.05) or LVL group (HVL 3.69 ± 0.36 vs. LVL 1.98 ± 0.48, p = 0.022; * < 0.05) but was no significant difference was found between LVL and Control group (LVL 1.98 ± 0.48 vs. Control 1.74 ± 0.40, p = 0.9213; * < 0.0332). (**b**) Representative figure of expression of NTCP in placenta through Immunohistochemistry, weak cytoplasmic staining in control, moderate to strong membranous and cytoplasmic staining in LVL and HVL group was observed. Strong membranous and cytoplasmic staining of non-pathological liver tissue was served as positive control. (**c**) IHC Scores were plotted against log of HBV DNA viral load (IU/ml) There was a significant difference between liver and control group (238 ± 44.88 vs. 65.5 ± 17.36, p = 0.0064; ** < 0.005) HVL and control group (HVL 185.5 ± 30.72 vs. Control 65.5 ± 17.36, p = 0.011; * < 0.05). Whereas, no significant difference was obtained between HVL and LVL groups (HVL 185.5 ± 9.71 vs. LVL 130.5 ± 30.45, p = 0.333) and LVL and control group (LVL 130.5 ± 30.45 vs. Control 65.5 ± 17.36, p = 0.30). *Con* Control, *HVL* High Viral Load Group, *LVL* Low Viral Load Group.

Nonetheless, presence of HBV replication marker in placental cells, led us to postulate that these cells express HBV receptor and we examined the expression of NTCP in the placental samples using qRT-PCR and IHC. Interestingly it was found that NTCP is expressed in placenta as well (Fig. 4). While the expression of gene encoding NTCP in LVL group was comparable to the control group (LVL 1.98 ± 0.48 vs. Control 1.74 ± 0.40, p = 0.9213; * < 0.05), it was significantly up regulated in HVL group when compared with control group (HVL 3.69 ± 0.36 vs. Control 1.74 ± 0.40, p = 0.0117; * < 0.05) or LVL group (HVL 3.69 ± 0.36 vs. LVL 1.98 ± 0.48, p = 0.022; * < 0.05). This suggest that NTCP expression might be associated with viral load (Fig. 5a). We further assessed the status of NTCP protein in placental cells using IHC. Expression of NTCP was observed mainly in trophoblasts while stromal and endothelial cells were not stained (Fig. 5b). Membranous and/or cytoplasmic staining was considered positive with reference to liver. There was a significant difference between liver and control groups (238 ± 44.88 vs. 65.5 ± 17.36, p = 0.0064; ** < 0.005) as well as HVL and control groups (HVL 185.5 ± 30.72 vs. Control 65.5 ± 17.36, p = 0.0131; * < 0.05). Whereas no significant difference was obtained between HVL and LVL groups (HVL 185.5 ± 30.72 vs. LVL 130.5 ± 30.45, p = 0.45) and LVL and control groups (LVL 130.5 ± 30.45 vs. Control 65.5 ± 17.36, p = 0.30) (Fig. 5c). Membranous expression was largely observed in HVL group placenta. For confirmation of specificity of NTCP antibodies we also analyzed the IHC of other tissues (Breast tissue, Myometrium and Endometrium of uterus) for NTCP expression and did not find it’s expression in any of these tissues (Supplementary Fig. 3). Presence of NTCP on placental cells further strengthen the argument that HBV can enter and replicate in these cells.

## Discussion

**Figure 6:**
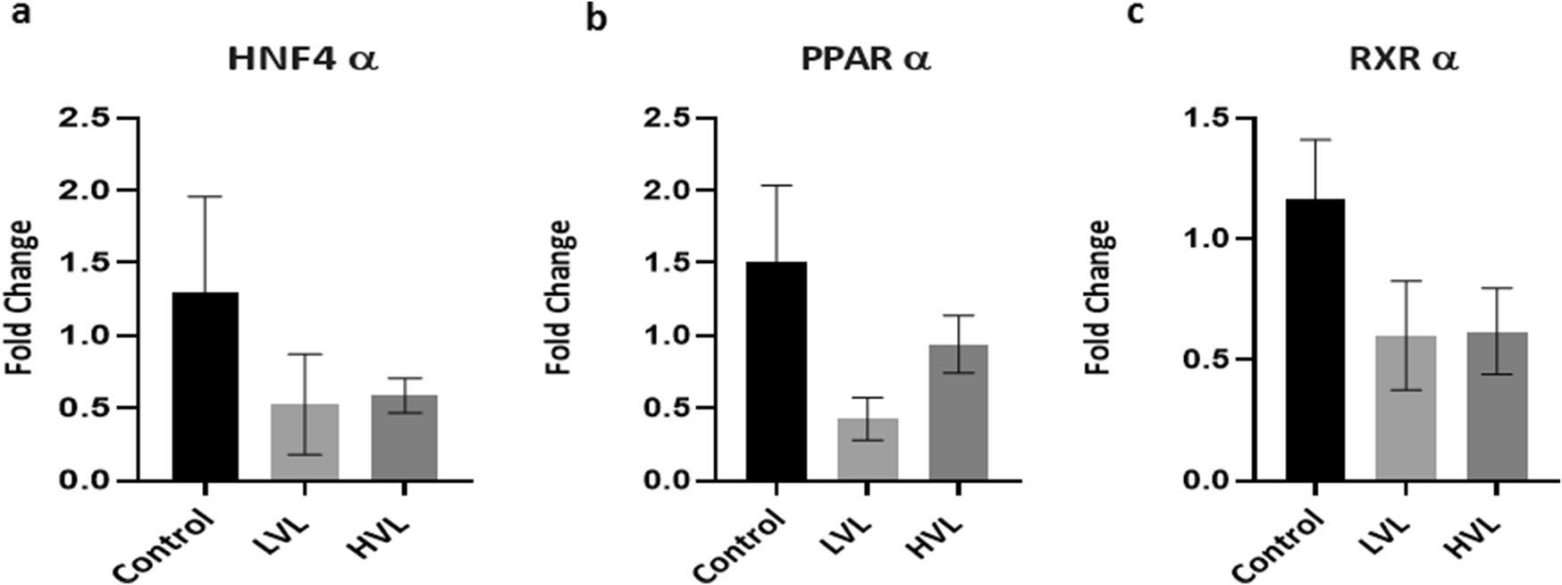
Differential expression of host transcription factors. mRNA levels of (**a**) HNF4α, (**b**) PPARα and (**c**) RXRα in placenta from the groups. *HVL* High Viral Load Group, *LVL* Low Viral Load Group, *HNF4α* Hepatocyte Nuclear Factor 4α, *PPARα* Proliferator Activated Receptor α, *RXRα* Retinoid X Receptor α.

While vertical transmission is the primary cause of chronic HBV infection worldwide, its mechanism(s) remain elusive. Expression of HBV receptor NTCP on placental cells and presence of the replication markers, cccDNA, pgRNA and HBeAg in placental cells provides a strong argument in favour of HBV replication in placenta. Host cells harbouring replicating HBV show a signature transcription profile with altered levels of host transcription factors such as Hepatocyte Nuclear Factor α (HNFα), Peroxisome Proliferator Activated Receptor α (PPARα) and Retinoid X Receptor (RXRα). These host transcription factors are reported to mediate replication of HBV in hepatocytes^13,14^. According to the “Human Protein Atlas” PAPRα and RXRα are reported in normal healthy placental tissue (trophoblasts). We checked the expression of these factors in the HBV infected placenta and these were expressed in the most of the samples analysed (Fig. 6).

**Figure 7:**
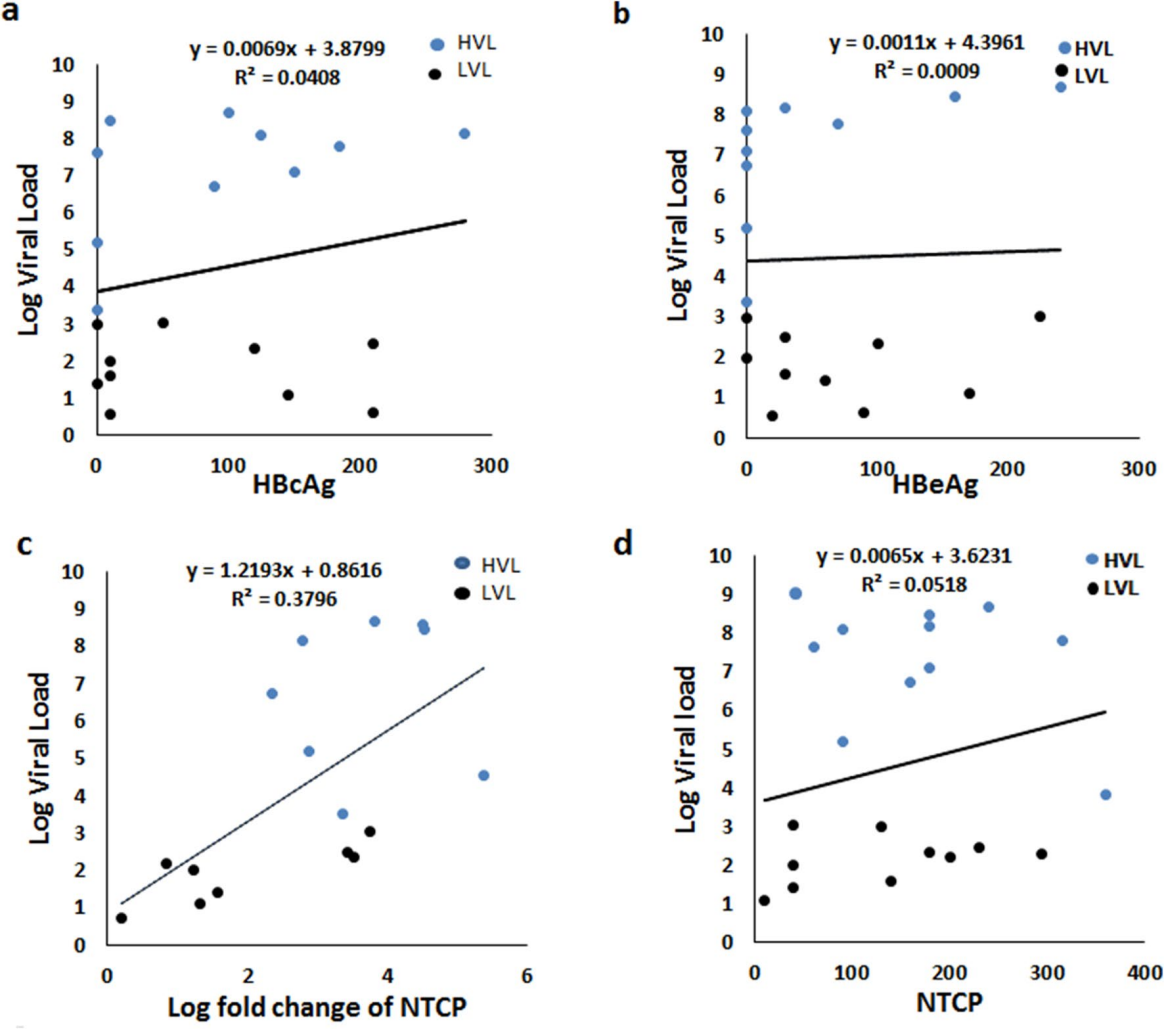
Dot plots showing correlation between, IHC scores of (**a**) HBcAg (PC = 0.04907, R^2^ = 0.0139) (**b**) HBeAg (PC = − 0.05213, R^2^ = 0.0009). (**c**) log fold change of NTCP expression (PC = 0.7118, R^2^ = 0.3796, p = 0.0027) and (**d**) IHC score of NTCP (PC = 0.3577, R^2^ = 0.0518) with viral load. Correlation coefficient was calculated using Spearman’s correlation test. *HVL* High Viral Load Group, *LVL* Low Viral Load Group.

In an earlier study, we demonstrated expression of the HBV entry factor Asialoglycoprotein Receptor (ASGPR)^6^. Expression of ASGPR together with NTCP on placental cells fulfils the binding and entry requirement, the precursor to HBV replication in placenta. It is a well-established fact that bile salt excretion is crucial for intrauterine life. Presence of NTCP along with other bile salt transporters on placental cells ensure this function. Other than that, NTCP may also play a role in transport of estriol precursor in placenta^15–17^. Presence of HBcAg and HBV DNA are the markers for infection and were reported in different tissues^9,18–20^. Several studies explored the transplacental route of intrauterine transfer. “Cellular transfer” was proposed by Xu et al. in 2002 evidenced by the presence of HBV DNA in placenta (in villous capillary endothelial cells) which decreases from mother side to the foetal side^21^. In 2007, Zhang et al. showed the presence of HBsAg and HBcAg in the different layers of placenta with decreases in concentration from maternal to foetal side^10^. The mechanistic evidences of transplacental transmission were given by Bhat et al., using BeWo (placental) cell line. They demonstrated the transcytosis of HBV in early gestation through microtubule dependent endosomal vesicles. They observed that temperature affected the endocytosis, leading them to postulate a possible receptor mediated entry of the virus. However, they did not investigate the presence of HBV receptors on these cells^7^. Later, other study also provides the cell models of trophoblasts to study the mechanism associated with transplacental route^22^. Though the previous studies provide evidences that HBV can cross the trophoblast barrier of placenta indicated by the presence of HBV DNA, HBcAg and HBsAg but the clear evidence of replication of HBV in placenta is lacking^23^. Previous studies also suggested that HBeAg can cross the placenta and associate it with vertical transmission but it was only based on the serological findings of mother and newborn^24,25^. In our study we did not find a clear relation between presences of HBeAg in placenta with the presence of this antigen in the peripheral blood half of the placenta samples from HBV positive females showed presence of HBeAg even in the absence of detectable peripheral antigen suggesting that placental HBeAg has resulted from local viral replication. Nonetheless, we acknowledge the possibility of cross reactivity of the antibody used for detecting HBeAg to HBcAg in tissue sections. To assess the specificity of the HBeAg antibody, we generated a heat map showing IHC scores of HBeAg and HBcAg and found that the samples strongly positive for HBeAg were mostly negative for HBcAg suggesting minimal cross reactivity, if any (Supplementary Fig. 4). We assessed the correlation between viral load in peripheral blood and the presence of HBcAg (PC = 0.6160, R^2^ = 0.0408) and HBeAg (PC = − 0.05213, R^2^ = 0.0009) in placenta and did not find any significant correlation (Fig. 7a,b). This observation further supports the argument of HBV replication in placenta.

We also assessed the correlation between expression of NTCP and viral load in peripheral blood. A significant positive correlation between log fold change of NTCP expression with viral load (PC = 0.7118, R^2^ = 0.3796, p = 0.0027) (Fig. 6c) was found although correlation was not significant when we compared IHC scores of NTCP (PC = 0.3577, R^2^ = 0.0518) (Fig. 6d) indicating probable post transcriptional modulation.

Despite multiple evidence indicate placental replication of HBV, very low level of cccDNA detected in the tissue suggests that unlike hepatocytes, placental cells support low level replication of the virus. A study with larger cohort along with the viral load measurements of cccDNA in placental sample will provide a better insight of this phenomenon. Other than this our study suffers from small sample size and lacks in vivo experiments, however, it does provide a reasonable basis to explore placental replication of HBV as this could aid in development of a significant strategy to curtail CHB infection.

## Conclusion

In this study, we for the first time provide experimental evidence to suggest the placental replication of HBV by demonstrating the presence of viral receptor, NTCP replication markers HBeAg, cccDNA and pgRNA in placental cells. Our findings provide an insight of a probable new mechanism contributing in vertical transmission of the virus and expected to be useful in developing therapeutic interventions.

## Materials and methods

### HBV pregnant females

A total of 27,407 pregnant females were screened for HBsAg during their antenatal routine check-up at a tertiary care and teaching hospital in central India over a period of 2 years from 2019 to 2020. A total of 432 of pregnant females were found positive for HBsAg (1.57%) and negative for hepatitis A, hepatitis C, hepatitis E and human immunodeficiency virus (HIV). None of the pregnant females had any systemic illness, autoimmune disease or inherited metabolic disorder. Out of 432, we were able to follow 59 HBsAg + ve pregnant females, till delivery due to loss of follow ups, lack of consent and other complications. Out of 59, 22 had HBV DNA ≥ 2000 IU/ml, 19 had HBV DNA < 2000 IU/ml and in 18 samples, HBV DNA was below the detection limit. A total of 41 HBsAg + ve and 10 healthy pregnant females were included in this study.

### Study groups

#### HBsAg positive mothers (n = 41)

HBsAg positive subjects were divided in two groups on the basis of viral load according to Indian National Association for Study of the Liver (INASL) guidelines 2018^11^. Pregnant females, with HBV DNA load ≥ 2000 IU/ml were categorized as High viral load (HVL) group (n = 22) and pregnant females, with HBV DNA load < 2000 IU/ml were categorized as Low viral load (LVL) group (n = 19).

#### Control group (n = 10)

Pregnant females, who were negative for HBsAg and gave birth to healthy new-borns, were identified as healthy and were included in the study as controls.

The study was conducted in accordance with the Declaration of Helsinki, and approved by the Institutional Human Ethics Committee of All India Medical Sciences (AIIMS), Bhopal, India (Approval No. EF0110 and EF0232). The Informed consent was obtained from each participant before enrolment in the study. The clinical and biochemical assessment of the subjects was done according to the study protocol. None of the participants was receiving anti-HBV drugs.

### Serological and virological studies

At term, approximately 5 ml of peripheral blood sample was collected in EDTA tubes and placental tissues were obtained from maternal decidua (within 6 h) of delivery. The plasma was separated within an hour of blood collection and immediately stored at − 80 °C till further use. HBeAg was quantified by ELISA using a kit (Dia. Pro Diagnostic Bioprobe, Italy) as per the instructions from the manufacturer.

### Peripheral HBV DNA quantification

HBV DNA quantitation was done with 500 μl plasma using TRUPCR^®^ HBV Viral Load Kit (3B Blackbio Biotech India Limited, Bhopal, India) as per the manufacturer’s protocol. This real-time PCR assay is based on dual labelled hybridization probe targeting the pre-core and core regions. Results were expressed as IU/ml. The lower limit of detection for the assay was < 2.5 IU/ml.

### DNA and RNA extraction from placental tissues

DNA and RNA were extracted from placental tissues frozen in RNA Liv (HiMedia, Thane, India). DNA from approximately 50 mg placental tissue was extracted using Hirt method^26^. Approximately 20 mg placental tissue was homogenised using TissueLyser LT (Qiagen) and total RNA was extracted using QIAamp^®^ RNA Blood Mini kit, (Qiagen, Germany) according to manufacturer’s protocol. DNA and RNA was quantified using NanoDrop™ Spectrophotometer (Thermo Scientific™). High-Capacity cDNA Reverse Transcription Kit (Applied Biosystems, Vilnius, Lithuania) was used for complementary DNA synthesis using 1 µg of RNA.

### Detection of HBV DNA in placenta

qPCR was performed with PowerUp™ SYBR™ Green Master Mix kit, (Applied Biosystems, Vilnius, Lithuania) using BioRad CFX96 Real Time PCR System (BioRad, Hercules, CA, USA) with primers specific for total HBV DNA targeting X and core ORF (HBV F: GAGGCTGTAGGCATAAATTGGTC, HBV R: AACTCCACAGAAGCTCCAAATTC). Every sample was independently analysed in triplicates.

### Detection of cccDNA in placenta

Two µg of isolated DNA was treated with 0.25 µl Exo I (NEB; 5 units) 0.25 µl Exo III (NEB; 25 units) and 0.5 µl T5 Exonuclease (5 units) 2 µl NEB Cutsmart buffer at 37 °C for 2 to 3 h in water bath. Control cccDNA was isolated from HepAD38 cells stably transfected with HBV DNA and makes cccDNA^11^. After enzyme digestion, to remove the nucleases, DNA was further purified by phenol–chloroform extraction and resuspended in 4 µl nuclease-free water.

For digital droplet PCR, 4 µl DNA was used in a total reaction volume of 20 µl with 10 µl of 2 × supermix and 1.5 µl primer–probe. The primers used for cccDNA-specific amplification were DR-F (GTCTGTGCCTTCTCATCTGC) (nt 1553 to 1572) and DR-R (ACAAGAGATGATTAGGCAGAGG) (nt 1830 to 1851) with target probe: 5′-FAM-CGTCGCATG-GARACCACCGTGAACGCC-BHQ1-3′ using QX100 ddPCR System (BioRad). To generate droplets, 20 µl of reaction mixture and 70 µl of droplet generation oil was placed in subsequent cartridge lanes and covered with gasket to place in the QX200 Droplet Generator (BioRad). Once the droplets were generated, 40 μl droplets from each sample were transferred into a 96-well plate and sealed with a sealer. Sealed plate was used for amplification using cycling condition; 95 °C for 10 min, followed by 40 cycles of 94 °C for 30 s and 60 °C for 60 s. Amplified products were read with a QX200 Droplet reader (BioRad). Absolute quantification (ABS) analysis was done by using QuantaSoft software^27,28^.

### Detection of pre-genomic RNA (pgHBV RNA)

cDNA was prepared using 100 ng of placental RNA from 3 samples of HVL group. HBV pre-genomic specific primers were used to prepare the cDNA (HBV F: GAGGCTGTAGGCATAAATTGGTC, HBV R: AACTCCACAGAAGCTCCAAATTC). cDNA with reverse transcriptase (RT) and without reverse transcriptase (NRT) was simultaneously prepared for each sample. The cDNA was subjected to qPCR using HBV pg primers and PowerUp™ SYBR™ Green Master Mix kit (Applied Biosystems, Vilnius, Lithuania) on BioRad CFX96 Real Time PCR System (BioRad, Hercules, CA, USA). GAPDH was used to assure cDNA synthesis. The amplicons obtained were confirmed by Sanger sequencing.

### Detection of HBcAg and HBeAg in placenta by immunohistochemistry (IHC)

Five-micron thick sections were cut from Formalin Fixed Paraffin Embedded (FFPE) tissue samples and deparaffinized by xylene followed by heat induced antigen retrieval by boiling at 100 °C for 10 min in citrate buffer (pH 6). Tissue sections were incubated with the following primary antibodies overnight at 4 °C: HBcAg (bsm-2000M; Bioss, 1:25), HBeAg (bsm-2022M; Bioss, 1:25). Polyexcel HRP/DAB detection system (PathnSitu, USA) was used for staining using manufacturer’s protocol and counterstaining was done with haematoxylin. Images were captured using Nikon DS-Ri2 camera on a Nikon Eclipse Ci microscope.

### Quantification of NTCP in placental tissues using qRT-PCR

cDNA prepared from 1 µg placental RNA was subjected to qPCR using PowerUp™ SYBR™ Green Master Mix kit (Applied Biosystems, Vilnius, Lithuania) on BioRad CFX96 Real Time PCR System (BioRad, Hercules, CA, USA). The specific exon spanning primers of NTCP were designed (NTCP Fwd: CTTCTGCCTCAATGGACGGT; NTCP Rv: GCCACATTGAGGATGGTGGA). qRT-PCR of every sample was done in triplicates to normalize NTCP expression level; GAPDH was used as reference gene. Subsequently, the relative gene expression values were determined using log of 2^−ΔΔCT^ where highest ΔCT within the group was used as calibrator to calculate ΔΔCT and specificity of product was assured by single melt peak.

### Detection of NTCP in placenta by immunohistochemistry (IHC)

Five-micron tissue sections were cut, and deparaffinized by xylene followed by heat induced antigen retrieval using boiling at 80 °C for 10 min followed by 100 °C for 10 min in Tris buffer (pH 9). Tissues were then incubated with the primary antibody anti-NTCP (BS1985R; Bioss, 1:1000) for 1 h at room temperature, further staining was done using Polyexcel HRP/DAB detection system (PathnSitu, USA) according to manufacturer’s protocol and were counterstained with haematoxylin. IHC was also performed on 5 non-HBV infected liver sections. Breast, endometrial and myometrial tissues were also stained and analysed. Images were captured using Nikon DS-Ri2 camera on a Nikon Eclipse Ci microscope.

### IHC quantification

IHC sections were scored by a pathologist using single blinded strategy. Membranous and/or cytoplasmic staining was considered as positive. IHC score was calculated using formula; IHC score = percentage of positive cells × score of intensity of the staining. The Score of Intensity followed 1—Weak, 2—Mild, 3—Moderate and 4—Strong for NTCP and HBcAg and 0—Weak, 1—Mild, 2—Moderate and 3—Strong for HBeAg. IHC score was used to calculate statistical significance.

### Detection of host transcription factors

cDNA prepared from placental RNA was subjected to qPCR using PowerUp™ SYBR™ Green Master Mix kit (Applied Biosystems, Vilnius, Lithuania) on BioRad CFX96 Real Time PCR System (BioRad, Hercules, CA, USA). The specific exon spanning primers for HNF4α, PPARα and RXRα were designed. (HNF4 α F: CGTGCTGCTCCTAGGCAAT, HNF4 α R: AAGGATGCGTATGGACACCC, PPARα F; GGATGTCACACAACGCGATTC, PPARα R; AGGCCTCGTAGATTCTCTTGG and RXRα F; GACGGAGCTTGTGTCCAAGA, RXRα R; GCCCCTTGGAGTCAGGGTTA). qRT-PCR of 6 samples from each group was done in duplicates. To normalize transcription factors’ expression level; GAPDH was used as reference gene. Subsequently, the relative gene expression values were determined using log of 2^-ΔΔCT^ where average ΔCT of control group was used as calibrator to calculate ΔΔCT and specificity of product was assured by single melt peak.

### Statistical analysis

All statistical tests were performed using GraphPad Prism for Windows version 8.0.1. Participant’s demographics are presented as median with range or mean with standard deviation. Categorical variables are presented as proportions while continuous variables are either presented as mean with standard error (SE) or median with range. Continuous variables were tested for normal distribution by Bartlett’s test followed by one way ANOVA test and Tukey’s multiple comparison test or Kruskal–Wallis test and Dunn's multiple comparisons test to find the significance. Correlation was calculated by Spearman’s statistics. All statistical comparisons were two tailed and a p value of < 0.05 was considered statistically significant.

## Supplementary Information

Supplementary Figure 1.

Supplementary Figure 2.

Supplementary Figure 3.

Supplementary Figure 4.

## Data Availability

The datasets generated during the current study are available from the corresponding author a88_ashish@yahoo.co.in on reasonable request.
