# Supplementary figures and images for: Presence of entry receptors and viral markers suggest a low level of placental replication of hepatitis B virus in a proportion of pregnant women infected with chronic hepatitis B
Source: Sci Rep. 2022 Oct 22;12:17795. doi: 10.1038/s41598-022-22699-8 (PMC9588053; doi:10.1038/s41598-022-22699-8)

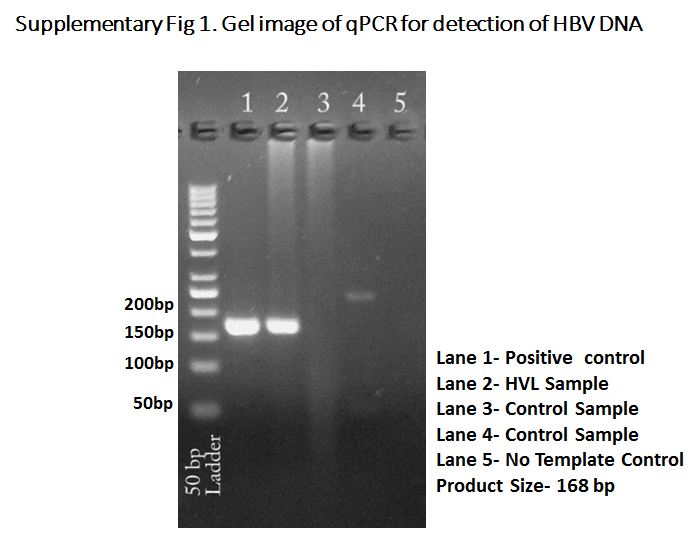

Supplement: Supplementary file 1 — Supplementary Figure 1. [file 41598_2022_22699_MOESM1_ESM.tif]

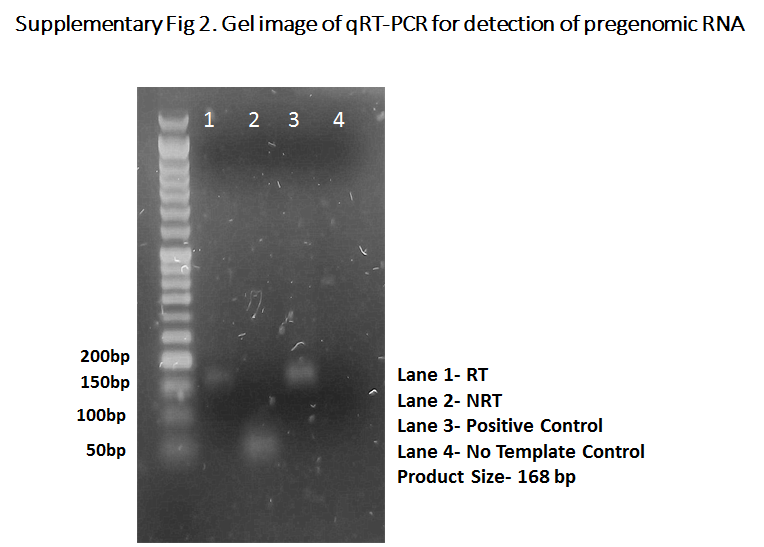

Supplement: Supplementary file 2 — Supplementary Figure 2. [file 41598_2022_22699_MOESM2_ESM.tif]

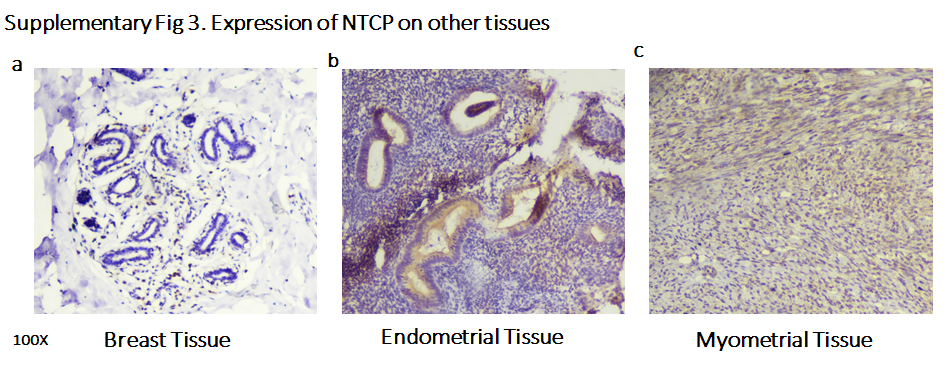

Supplement: Supplementary file 3 — Supplementary Figure 3. [file 41598_2022_22699_MOESM3_ESM.tif]

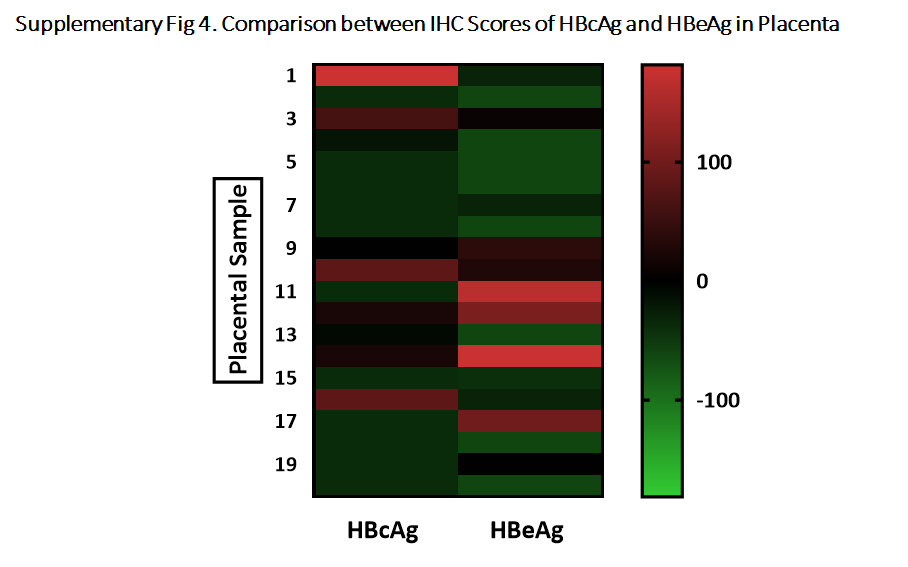

Supplement: Supplementary file 4 — Supplementary Figure 4. [file 41598_2022_22699_MOESM4_ESM.tif]
